# Supplementary material for: Artificial intelligence based writer identification generates new evidence for the unknown scribes of the Dead Sea Scrolls exemplified by the Great Isaiah Scroll (1QIsaa)
Source: PLoS One. 2021 Apr 21;16(4):e0249769. doi: 10.1371/journal.pone.0249769 (PMC8059865; doi:10.1371/journal.pone.0249769)
Supplement: S1 File — (PDF) [file pone.0249769.s001.pdf]

All supplementary materials (S1, S2, S3, S4, and S5) for the article:

Artificial intelligence based writer identification generates new evidence for the unknown scribes of the Dead Sea Scrolls exemplified by the Great Isaiah Scroll (1QIsa^{a})

Mladen Popović[1◕*], Maruf A. Dhali [2◕], Lambert Schomaker[2◕]

1 Qumran Institute, Faculty of Theology and Religious Studies, University of Groningen, Groningen, The Netherlands
2 Department of Artificial Intelligence, Faculty of Science and Engineering, University of Groningen, Groningen, The Netherlands

◕ These authors contributed equally to this work.

* Corresponding author's address: Qumran Institute, Oude Boteringestraat 38, 9712 GK Groningen, The Netherlands. Email: m.popovic@rug.nl

S1 Supposed scribal idiosyncrasies

The study in [1] suggests that there are nine scribal idiosyncrasies in both halves of the manuscript. Since almost all of the features concern scribal practices shared also by other scribes in the scrolls, the nine features listed by [1] are not scribal idiosyncrasies and therefore do not support there being one main scribe in 1QIsa^{a}:

- For other examples of writing of parts of words at the end of a line to be repeated in full on the following line for lack of space, see [2], 107–108;
- For other examples of supralinear and infralinear writing at the end of a line, see [2], 108. Also, 1QIsa^{a} columns 3 and 30 simply did not allow extending much beyond the left column margin because of the stitches connecting two sheets (and in column 45:10 the intercolumn space was already used up);
- For a discussion of extending beyond the right column margin, see [2], 106;
- Regarding the ligature *samek* and final *pe* occurring virtually only in כסף:

  1. There are seven examples where this does not occur (cf. 1QIsa^{a} 2:16; 32:17; 33:19; 39:11; 43:17; 45:19; 45:20) and five examples where it does occur (cf. 1QIsa^{a} 7:14; 37:3; 40:15; 45:19; 49:20 [the last one is not listed by [1]]), sometimes in the same line (45:19);

  2. The way in which *samek* and final *pe* are written in 1QIsa^{a} 7:14 differs from the other four examples from the second half of the manuscript (especially in the horizontal upper stroke of *samek* and in the horizontal down stroke of final *pe*), whereas those four are written in the same way;

  3. All other occurrences of words with *samek* and final *pe* are non-ligatured except for יאסף in 1QIsa^{a} 49:23;
- For another example of starting to write the *lamed* too soon, see 4Q27 15:10;
- For many more examples of crossing out words or letters, see [2], 198–201;
- For a discussion of many other examples of cancellation dots, see [2], 188–198.

S2 Image information

Table 1. Brill scan numbers, Shrine of the Book (Israel Museum, Jerusalem) inventory numbers, and their corresponding column numbers for 1QIsa^{a}.

Scan number	Column	Scan number	Column	Scan number	Column
2162/SHR 7001	**1**	2181/SHR 7022	**22**	2209/SHR 7043	**43**
2163/SHR 7002	**2**	2182/SHR 7024	**24**	2210/SHR 7044	**44**
2164/SHR 7003	**3**	2183/SHR 7026	**26**	2211/SHR 7045	**45**
2165//SHR 7004	**4**	2184/SHR 7027	**27**	2212/SHR 7047	**47**
2166/SHR 7005	**5**	2185/SHR 7028	**28**	2213/SHR 7048	**48**
2167/SHR 7006	**6**	2186/SHR 7029	**29**	2214/SHR 7049	**49**
2168/SHR 7007	**7**	2187/SHR 7030	**30**	2215/SHR 7050	**50**
2169/SHR 7008	**8**	2188/SHR 7032	**32**	2216/SHR 7051	**51**
2170/SHR 7009	**9**	2189/SHR 7033	**33**	2217/SHR 7053	**53**
2171/SHR 7012	**12**	2190/SHR 7034	**34**	2449/SHR 7010	**10**
2172/SHR 7013	**13**	2191/SHR 7035	**35**	2450/SHR 7011	**11**
2173/SHR 7014	**14**	2192/SHR 7036	**36**	2451/SHR 7023	**23**
2175/SHR 7015	**15**	2193/SHR 7037	**37**	2452/SHR 7025	**25**
2176/SHR 7017	**17**	2194/SHR 7038	**38**	2453/SHR 7031	**31**
2177/SHR 7018	**18**	2195/SHR 7039	**39**	2455/SHR 7052	**52**
2178/SHR 7019	**19**	2196/SHR 7040	**40**	2456/SHR 7054	**54**
2179/SHR 7020	**20**	2197/SHR 7041	**41**	-	-
2180/SHR 7021	**21**	2208/SHR 7042	**42**	-	-

S3 Primary analyses

S3.1 Preprocessing: Binarization and alignment correction

Our first step of preprocessing was binarizing the images of 1QIsa^{a}.

It should be noted here that many modern deep-learning methods can be trained end-to-end with the 1QIsa^{a} scroll without performing binarization, but this is not desirable for doing digital palaeography of the scroll. For example, a direct end-to-end solution on clustering the column-images of the 1QIsa^{a} scroll can be achieved for writer identification, but there is always a risk of getting the solution for the wrong cause. For example, the decision of an artificial neural network may be based on spurious correlations with the texture of the parchment. Therefore, it is essential to extract only the ink traces (foreground) and no other material features in the images (background). There are several traditional methods for document binarization. Most commonly used ones are Otsu [3] and Sauvola [4]. These traditional methods work quite well if the contrast between the ink and the background is relatively large. But for the 1QIsa^{a} images this is not the case mostly due to degradation over time and the skin texture. It is, therefore, important to digitally extract the ink from the parchment. In this study, we use BiNet, a deep-learning-based method especially designed in Groningen to binarize the scrolls images. Instead of using a simple filtering technique, BiNet uses a neural network architecture for the binarization task and therefore yields better output [5].

After performing the binarization, the images need to be cleaned further. This cleaning is required to get rid of the adjoining columns that appear in each of the images of the target columns. This is an important step as it will ensure that every image corresponding to a particular column should contain the characters from that target column only. Following the cleaning process, rotation and alignment correction needs to be done as well. If the images are rotated with an angle to the horizontal axis, then this affects the feature calculations that are not rotation-invariant. The rotation correction will thus ensure that the lines of texts are aligned horizontally. After this step, in a few cases, minor affine transformation and stretching correction is performed in a restrictive manner. These corrections are also targeted for aligning the texts where the text lines get twisted due to the degradation of the parchment (Fig. 2 in the main article). For the rotation and stretching correction, we have used the well-known GIMP tool, a free and open-source raster graphics editor (version 2.8.16) [6]. Finally, we have split each of the columns vertically into half to further validate the tests.

S3.1.1 Image morphing: Adding random noise to the data

During the writing process of any document, a writer naturally introduces variability. In contrast to within-writer variability, there are differences (variations) among writers. In order to address this issue, we introduced noise to the data. The noise will, on the one hand, ensure the system's robustness and, on the other hand, take into account variations within and between writers to notice the change in the outcome. Our approach is to take the columns of 1QIsa^{a} and augment them by generating synthetic images using random geometric distortions [7].

We add noise to the data by applying random elastic 'rubber-sheet' transforms. For each pixel (i, j) of the column images, a random displacement vector $(\Delta x, \Delta y)$ is generated. The complete image's displacement field is smoothed using a Gaussian convolution kernel with a standard deviation σ. We then rescale the field to an average amplitude A. The new morphed image (i', j') is generated using the displacement field and bilinear interpolation:

$$i' = i + \Delta x, j' = j + \Delta y. \quad (1)$$

Two parameters control this morphing process: the smoothing radius σ and the average pixel displacement A. Both parameters are measured in units of pixels. In our experiment, we empirically chose a displacement value of 1.0 and a smoothing radius of 8.0. Figure 1 shows an example of original image and morphed images for column 15 of 1QIsa^{a}. We perform morphing on both full and half-split columns. Together with the original ones, these morphed data provide us with a large number of images from 1QIsa^{a} to test with our system. We perform the same procedure from the primary and secondary analyses to all the

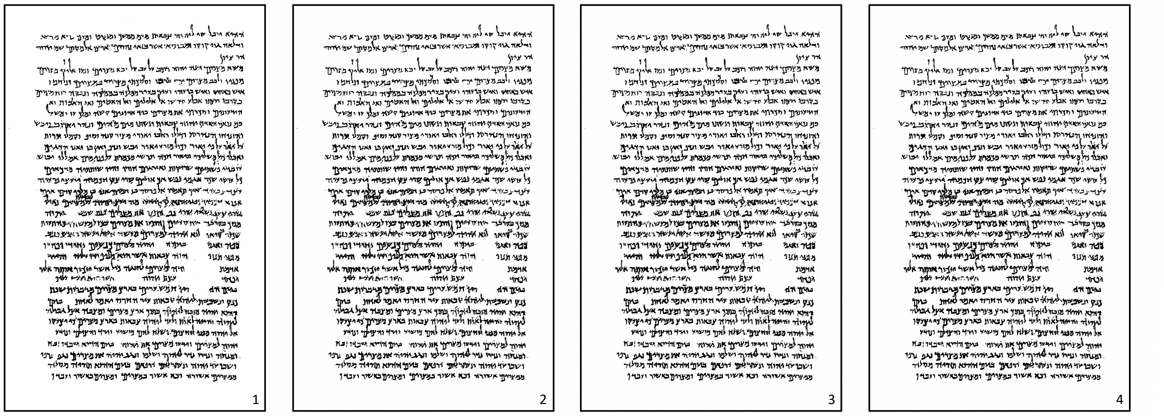

Fig 1. *(from left to right)* The original binarized image of column 15 and three randomly augmented morphed images. A close inspection of the images shows small geometric distortion introduced to the characters using the elastic-morphing technique.

newly generated augmented data. The results from the augmented data show no notable change in the original finding.

S3.1.2 Feature extraction: Texture-level

Textural methods capture statistical information on attributes of handwriting, like the curvature and slant of the contours of characters. As these methods look at the image as a whole, they do not require a dedicated segmentation technique. The statistical information in the feature vector represents the handwriting style of the document to be used in further analysis. As mentioned above, Hinge is a successful textural feature-extraction technique for the scrolls collection and we use it for 1QIsa^{a} in our current study. Hinge is originally proposed in the work of Marius Bulacu and Lambert Schomaker [8].

The Hinge feature is a compact transformation of the handwriting that captures both the writing angle and trace curvature. The bio-mechanics (relative wrist/finger movement control) and allographic choice (the learned and preferred letter shapes) of the writer dictates the slant and roundness of the writing process. As Hinge captures these two basic parameters (slant and roundness), it comfortably succeeds in writer identification and verification.

The Hinge kernel calculates the joint probability distribution of the angle combination of two hinged edge fragments. The joint probability of the orientations is quantized into a two-dimensional histogram $p(\alpha, \beta)$, where the angles α and $\beta (\alpha < \beta)$ are the angles with respect to the horizontal plane, of the two arms of a hinged kernel that is convolved over the edges of a handwritten image. For actual calculations, the hinge can be slid along the contour of each connected ink component of writing. We use 31 angles ($nbins$: number of bins) for both α and β with a length ($nvec$) of 13 (Fig.2). We only consider the angles that are smaller than 180° (to get rid of redundancy), and we can exclude the cases in which $\alpha == \beta$ (this is because if they are equal than it implies that they are indicating the same point and there is no useful angle involved). Finally, it results in a feature vector of 465 dimensions.

S3.1.3 Feature extraction: Allograph level with neural networks

The next type of feature we use is on the character-shape (allograph) level, namely the Fraglet. We will briefly explain how the Fraglets are formed. The connected components (mostly the full character shapes) from binarized images are fragmented to get more prototypical shapes from the scrolls collection.

Each fragmented contour (counter-clockwise traced) contains 200 points yielding 400 feature values (x, y: position of each pixels). The contours are normalized to a centre of gravity at $(0.0, 0.0)$, with the radius emanating from that centre being normalized to an average of $r = 1.0$. This type of normalization is more stable than bounding-box normalization (Bounding-boxes refer to minimum rectangles containing

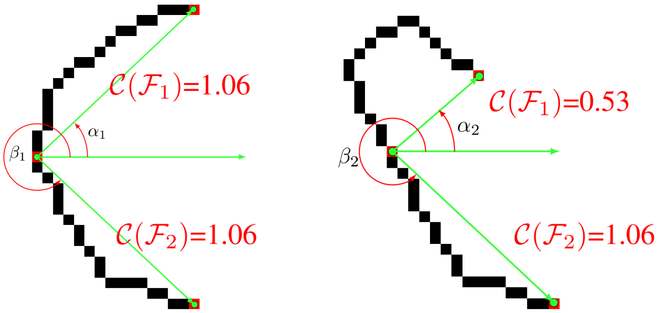

Fig 2. Hinge kernel; the angles and leg-lengths for two different character shapes.

the ink pixels of individual characters. They create more difficulties in normalization due to different and often arbitrary shapes of ink blobs.). We call these contours the Fraglets.

In order to extract these Fraglets, we used binarized images from the scrolls collection with the condition that the images need to have at least $100,000 (100k)$ black pixels. This ensures automatic selection of images with a relatively high amount of writing. Finally, 746 full plate images from the scrolls collection fulfilled this criterion. For the full plate images, we use the high-resolution multi-spectral images kindly provided to us by the Israel Antiquities Authority (IAA), which derive from their Leon Levy Dead Sea Scrolls Digital Library project [9].

Using the extracted Fraglets, we than form a Kohonen Map. As mentioned above, this is a self-organizing feature map (SOFM) that uses neural networks with neighbourhood function to preserve the topological properties of the Fraglets. The resulting SOFM contains $70x70$ cells with each cell containing 400 features (Fig. 3). The number of cells in the SOFM is derived empirically by measuring the performance on the writer identification data from our previous study on the scrolls [10].

Once the Kohonen Map (SOFM) is formed, we then use it to calculate the Fraglet feature for 1QIsa^{a}. For each of the images of the columns, we calculate a feature vector of the output histogram. We take a spread of counts for a Fraglet over 30 nearest neighbours in the SOFM. We also calculate the cosine feature (and corresponding cosine SOFM file). This involves replacement of the normalized coordinates with cosine/sine pairs. This means (x, y) coordinates become $(cos(\phi), sin(\phi))$ with phi representing the angle with the horizontal axis, for each coordinate point along the contour. Finally, it results in a feature vector of 4900 dimensions.

S3.1.4 Adjoined Feature

In order to take advantage from both the textural and allographic feature level, we use a third type of feature namely the Adjoined features. Adjoined features are the weighted combination of both Hinge and Fraglet. The adjoining results in a feature vector of 5365 dimensions preserving the handwriting style description from both feature levels.

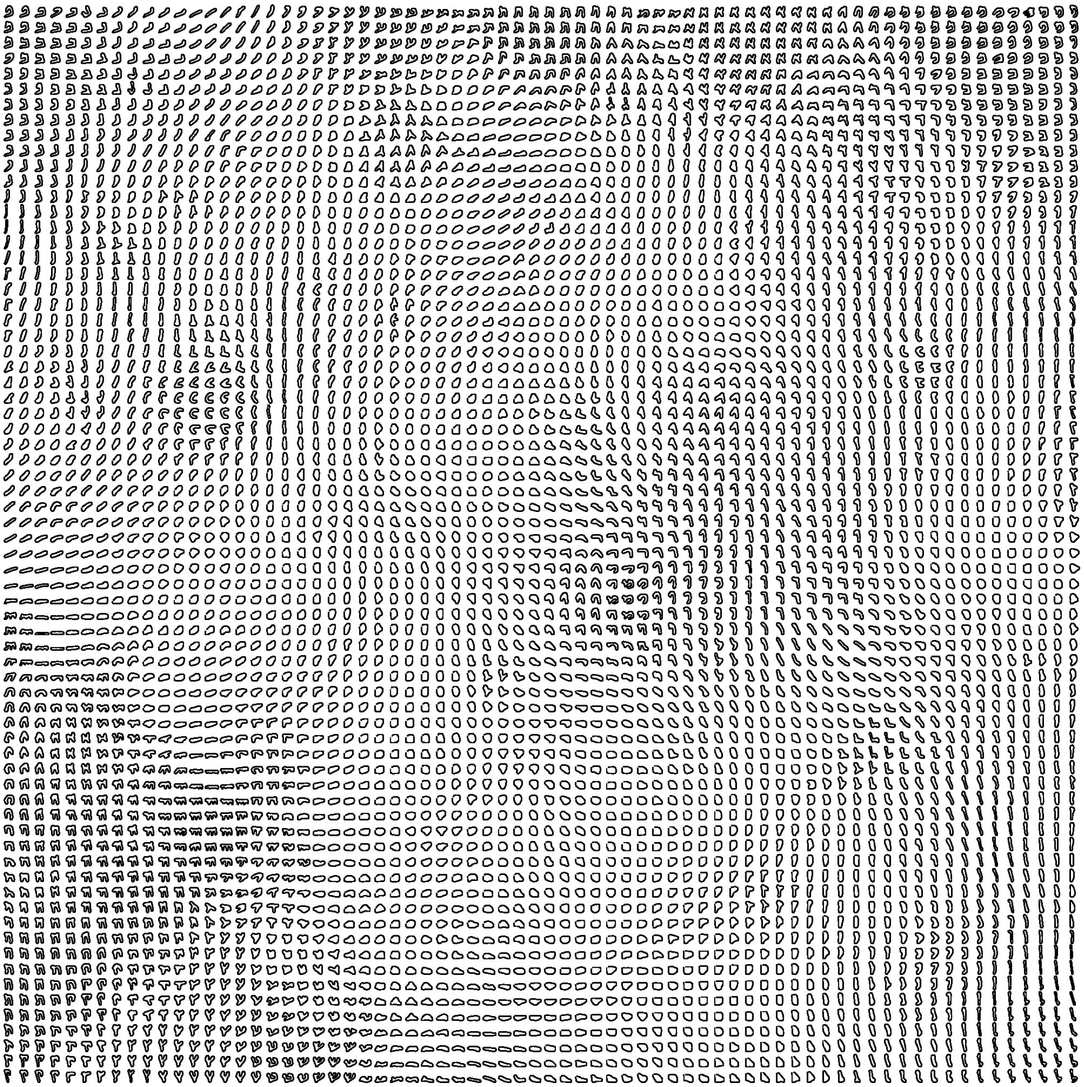

Fig 3. A visualization of 70x70 Kohonen SOFM using Fraglets from the DSS collection.

S4 Secondary analyses

S4.1 Kohonen map of fragmented connected components

In the so-called bag-of-patterns approach used here, a document is assumed to be characterized by the usage (occurrence) frequencies, i.e., the histogram of the fraglets, similar to the well-known bag-of-words approach in text analysis [11]. The distance between such histograms is computed for pairs of document samples. The histogram is assumed to be a feature vector capturing the occurrence of small, prototypical shapes, such that an overall descriptor for the style of each document sample can be computed. Fraglets do not have to correspond to complete characters, they can be smaller or larger than that, and each is mapped to its best-matching centroid in the SOFM, which is guaranteed not to represent an outlier or singleton pattern due to the very large size of the training data.

Figure 4 shows the complete SOFM that was computed, separately from the map that was used in the primary exploratory analysis, on the basis of $600k$ fragmented connected contours derived from binarized IAA images. Figure 5 shows an enlarged portion of the total map.

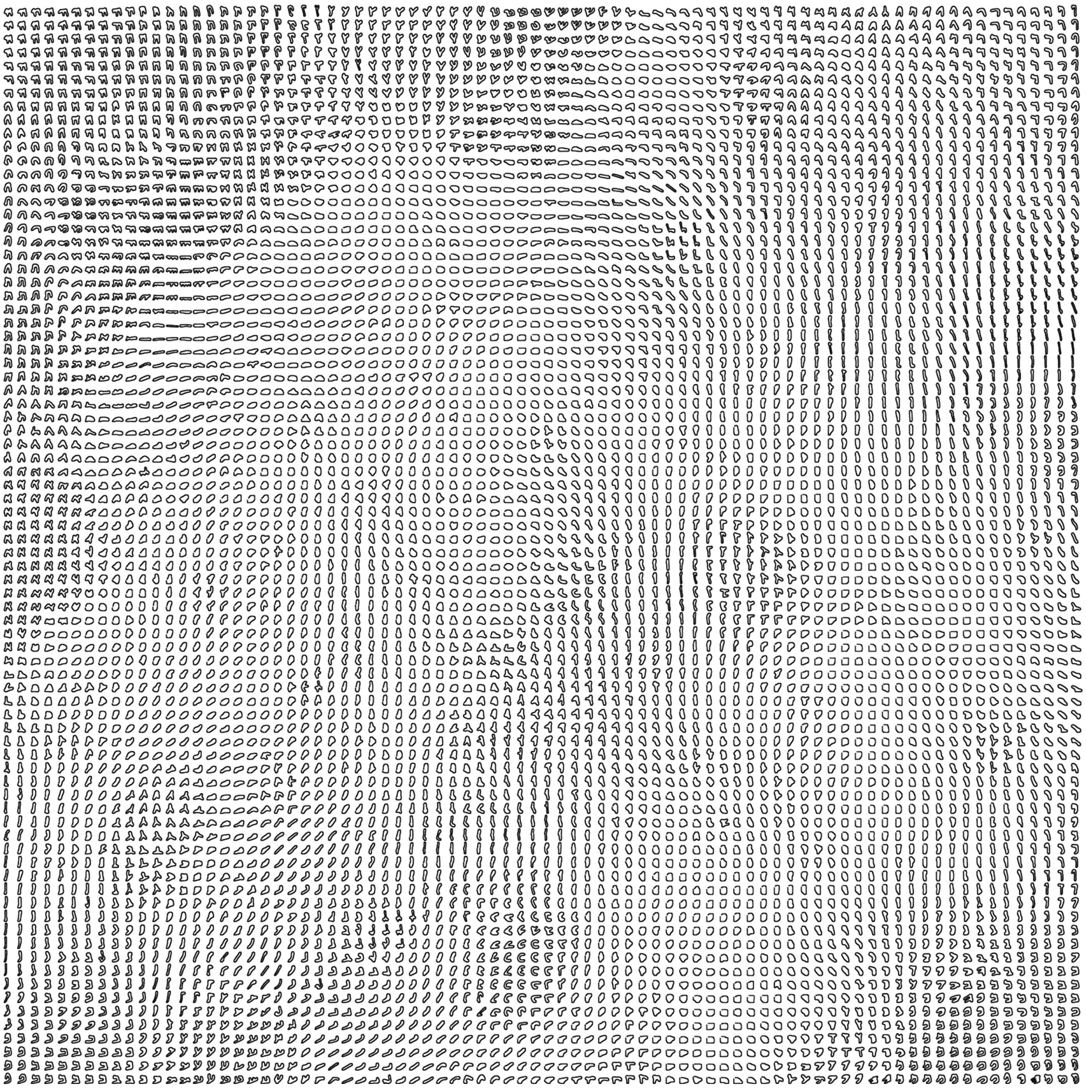

Fig 4. 80x80 Kohonen map of fragmented connected components (200 x,y points per contour centroid).

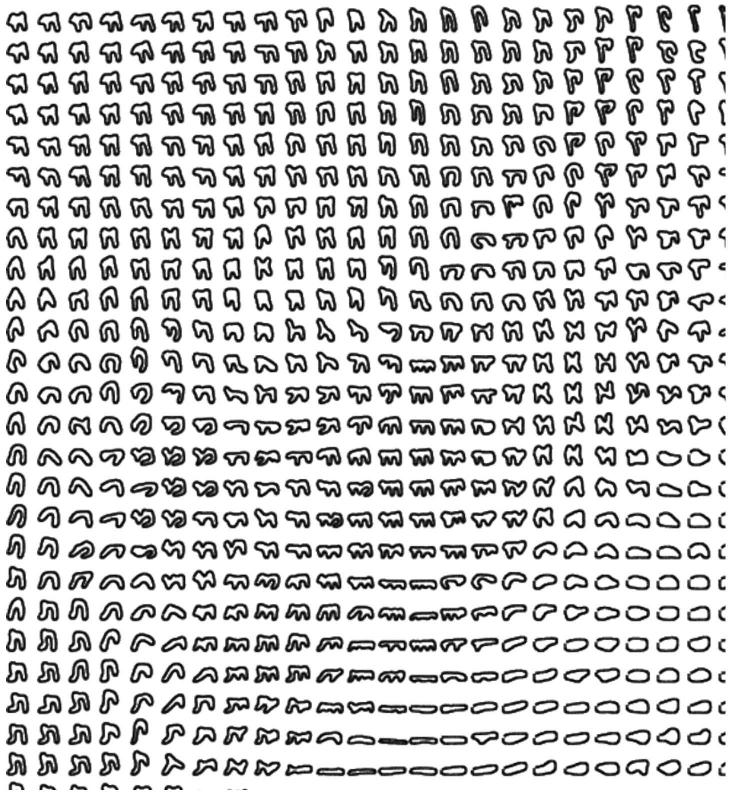

Fig 5. Portion of 80x80 Kohonen map of fragmented connected components.

S4.2 Statistical tests on the fraglet feature distances

Step 5a - If the style were uniform, there should be no difference between the number of times a hit (nearest neighbour) is found on the left or on the right of a column number. Using the Chi-square test, the deviation from the expected frequencies can be computed. If it is more likely that a point on the left has a hit on the left in the sequence, or, vice versa, finding a hit on the right of a point that is on the right, then the distribution is not homogeneous. The window under consideration in the column series is varied from 9 to 26: big enough to catch hits, but smaller than the mid point of the sequence. The returned probability that the pattern of counts is non-accidental will be averaged and a graph will be plotted over the column numbers. A minimum or dip in the curve will be indicative of a column number where the voting pattern for left vs right column hits is not random. The common threshold of $\alpha = 0.05$ will be used to decide for such singular points. No information concerning a critical column number is used. Due to the dependent nature of the running time window of left and right votes for neighbours, additional testing is needed.

Step 5b - If the style were uniform over columns, the distances to the nearest neighbours on the left and right should be comparable (of similar value) over the column series. On the other hand, if there are style differences, the average value of the distance may change over the column series. For this, a one-way analysis of variance can be used, or a t test, with the categories left and right, for the leftmost and rightmost columns in the series, respectively. Also here, a windowed approach is used, where distances are computed over windows of size 18 to 26 columns and averaged. Please note that, similar to the approach in Question 5a, no information is used concerning a column where style transition may be supposed to occur.

F-test: An F-test is used for a ratio of two variances, assuming $alpha = 0.05$ as the threshold. From all the input data, only $nth = nth_1$ is selected, i.e., the best nearest neighbour. Assuming column-a and column-b samples for the series, we select all cases where the nearest neighbour is

‘left’ when the target is ‘left’, and the nearest neighbour is ‘right’ when the target is ‘right’. We then compute the distance variances between each target and its best neighbour for both left and right.

```
F is varright/varleft: 1.77731
nleft=40 nright=44
```

Note that the numbers *nleft* and *nright* already indicate that ‘left’ looks predominantly like left (40 out of max count: 52) and ‘right’ looks predominantly like right (44 out of max count: 52). And, F-threshold ($alpha = 0.05$) is 1.701 and the obtained number is 1.77731 (larger; further confirms the claim of left and right).

```
Subtract 1 to obtain the degrees of freedom:
ndf: 39 vs 43.
Result of the F-test:
varleft=3.56642e-05
varright=6.33863e-05
F=1.77731
ndf: nleft=39 nright=43
varright/varleft:  p < 0.04  (is < alpha=0.05)
```

Step 5c - If the style were uniform, we would expect the same average position for hits over the column series. Indeed, the average position should be in the middle of the column series. On the other hand, if there are style differences, the average estimated position per column would vary. In the case of a linear style development in the series, the estimated average position of hits would also vary linearly. If a sudden change in style occurs, alternatively, we would expect something like a ‘step response’, i.e., a discontinuity in the series.

Step 5d - Following 5c, if there is a phase transition in the sequence of columns, fitting a logistic curve on the variable ’average neighbour position’ over columns should reveal the switching point reliably, i.e., with a high Pearson correlation of the fit. The number of the critical phase-transition column is the output of this test.

```
--------------------------------------------------------
One-way ANOVA
Name              N     Mean       SD       Min       Max
Left             18    0.238    0.003    0.233    0.243
Right            17    0.231    0.008    0.220    0.249
Total            35    0.234    0.007    0.220    0.249
--------------------------------------------------------
Weighted Means Analysis:
Source           SS    df         MS        F        p
Between       0.000     1      0.000   11.189    0.002 **
--------------------------------------------------------
t-test
Name           N     Mean       SD       Min       Max
Group-1       18    0.238    0.003    0.233    0.243
Group-2       17    0.231    0.008    0.220    0.249
Total         35    0.234    0.007    0.220    0.249
--------------------------------------------------------
Weighted Means Analysis:
t(33) = 3.345   p = 0.002
```

The distance of a column with a nearest neighbour is significantly different between matches found to the left and the right ($p < 0.005$), with a slightly larger distance for the first half ($d = 0.238$) as compared to the second half ($d = 0.231$). One-way anova and a t-test both return $p = 0.002$.

The average distances obtained if the queried column is on the left of the found column in the sequence (best hit is in the future). Between column 25 and 30, this distance drops, i.e., 'future' columns fit better. After column 35, the distance increases again.The mirror version, i.e., 'best hit for a query is in the past' also shows a transition between column 25 and 30. The pattern is a bit less clear but confirms the notion of an accident. Average column position of the best fitting neighbour for a column. Average position of best-fitting neighbours of a column, in the column series. Left of column 27, the average position of the hits is between 20-25. On the right of column 27, the average position of hits is between 30-35. The light blue line represents column 27. In case of a linear style development, the diagonal blue line should have been approximated. In case of no style development, the y-values should have been about constant.

One-way ANOVA for variable: position of nearest neighbour (column number) of a given DSS column. The nearest neighbour is computed in fraglet-histogram space ($Ndim = 6400$), for two groups: left (column <= 27) and right (column > 27).

```
---------------------------------------------------------
Name           N     Mean       SD      Min      Max
LEFT          27   23.847    4.008   16.312   35.688
RIGHT         27   32.023    3.689   25.625   38.688
Total         54   27.935    5.620   16.312   38.688
---------------------------------------------------------
Weighted Means Analysis:
Source            SS    df         MS        F     p
Between      902.418     1    902.418   60.822 0.000 ***
Within       771.519    52     14.837
---------------------------------------------------------
Again: p < 0.001
```

Also this analysis indicates that the between-column similarity is highest 'ipsilateral' with respect to the cut point (column 27): left looks like left, right looks like right. The significance is so high that with three decimals, p appears as zero in the output of the statistics tool. We can safely say that $p < alpha = 0.001$. This is a rigorous alpha, similar to medical sample comparisons, i.e., a three stars result (***). In other words: the probability that this difference is the consequence of random fluctuation, is less than $p = 0.001$.

- The midpoint for category 'left' is at column 24.
- The midpoint for category 'right' is at column 32.

S4.2.1 Finding the phase transition using a logistic fit

Assumption: in the column series there is a phase transition somewhere in the series. Indications for this came from Chi-square tests on the distributions of hits left—right of a target column. These tests indicates a switch at around column 27. When using this as the split criterion, a subsequent one-way anova revealed significant difference p ¡ 0.001 for columns on the left and right, who appear to have their nearest-neighbour hits on the left and right, respectively, consistent with the expectation of large similarity within a grouping. If we model the series as a phase transition, using a logistic function, will that transition occur at or about column 27?

Without seeding a logistic function estimator with knowledge concerning the magical number 27, this was the output:

```
-----------------------------------------------------------------
                        xoff      yoff      amplitude  steepness
mc-logist-reg-predict   27.824583 24.094666 7.983507   0.924704
-----------------------------------------------------------------
```

The value of xoff means that the transition column is estimated to occur between column 27 and 28, with a transition steepness that is smooth: In the separate .svg plot it lasts from 24-32.

Sigmoid regression analysis output:

```
-----------------------------------------------------
Analysis for 54 cases of 2 variables:
Variable      sigmoid     avgpos
Min           24.0947    16.3125
Max           32.0782    38.6875
Sum         1514.0753  1508.5000
Mean          28.0384    27.9352
SD             3.8642     5.6199
-----------------------------------------------------
Correlation Matrix:
sigmoid        1.0000
avgpos         0.7432     1.0000
Variable      sigmoid     avgpos
-----------------------------------------------------
Regression Equation for sigmoid:
sigmoid  =  0.511 avgpos  +  13.7632
-----------------------------------------------------
Significance test for prediction of sigmoid
    Mult-R  R-Squared      SEest    F(1,52)    prob (F)
    0.7432     0.5523     2.6102    64.1608     0.0000
-----------------------------------------------------
```

The fit of the sigmoid transition model is significant, with a correlation r that equals 0.74 (p ¡ 0.001). An exact fit would have yielded r=1.0. Although not perfect, r=0.74 would be considered as a very robust correlation in psychology and biology.

The model would explain 55% of the variance in the data, which is not strange, given the fact that the model is a stylized description of a time sequence with irregularities. If we smooth the irregularities over time, using a running average over a limited 3 or over 5 samples (columns), to smooth out the within writer variation, the correlation with the sigmoid increases considerably:

- If we smooth the column time series over 3 values, r=0.87 (76% var. explained variance by sigmoid phase transition)
- If we smooth the column time series over 5 values, r=0.93 (86% var. explained variance by sigmoid phase transition)

S4.3 Least-squares fitting (Scipy) of a logistic curve on the estimated average serial position of the nearest-neighbour of a column

The result of the Monte Carlo-based logistic model fit was replicated with a more traditional least-squares curve fit (Python scipy), yielding a phase transition at column 26.6 for raw data, with r=0.74, at 26.2 for a smoothed time series with a window of three points (r=0.87) and a transition at column 26 for a smoothed time series with a window of five points (r=0.94). Curve-fitting results are shown in Fig. 6, 7 and 8. Even without smoothing, the phase transition is clearly visible. With smoothing, the pattern is even more clear (window size 5, Fig. 8).

```
----------------------------------------------------------------
Raw Monte Carlo (mc:) and least squares (scipy:) results
----------------------------------------------------------------
  Xoffset     Yoffset   A       steepness
 27.824583 24.094666 7.983507 0.924704 < mc:try     r=0.7432
 28.243372 24.213305 7.994352 2.602412 < mc:RAW     r=0.7363
 27.727867 23.932599 8.002640 0.876055 < mc:RAW     r=0.7432
 27.634910 24.354421 7.740445 1.295146 < mc:RAW     r=0.7414
 27.882597 24.194399 7.872786 7.975232 < mc:RAW     r=0.7365
 26.605149 23.233309 9.102090 0.435106 < scipy:RAW  r=0.7441
----------------------------------------------------------------
 27.9197 Avg,RAW
 27.170642 23.515902 7.901666 0.751979 < mc:SMO3    r=0.8692
 25.067162 22.548375 9.256635 0.362236 < mc:SMO3    r=0.8713
 24.622219 22.198244 9.320780 0.468216 < mc:SMO3    r=0.8665
 28.309579 23.505822 8.146873 1.383725 < mc:SMO3    r=0.8674
 26.210565 23.064034 9.189271 0.407289 < scipy:SMO3 r=0.8728
----------------------------------------------------------------
 26.2924 Avg,SMO3
 25.907792 23.278395 8.809249 0.572107 < mc:SMO5    r=0.9316
 23.054408 22.920690 8.547156 0.335815 < mc:SMO5    r=0.9243
 24.057952 22.826461 8.236999 0.341474 < mc:SMO5    r=0.9309
 24.405605 22.058579 9.809585 0.280687 < mc:SMO5    r=0.9319
 25.990002 22.951100 9.342036 0.389834 < scipy:SMO5 r=0.9360
----------------------------------------------------------------
 24.3564 Avg,SMO5
```

The least-squares approach gives one result. The Monte Carlo estimation is done a few times (1 hour of computing per fit). Although smoothing over 5 columns (SMO5) gives the highest value of the Pearson correlation, the smoothing also biases the estimation of the transition point. Therefore the estimate of the transition point for 'RAW' data is to be preferred. The estimation yields a negative number for the Xoffset, this corrected here, to be consistent with Eq. 1.

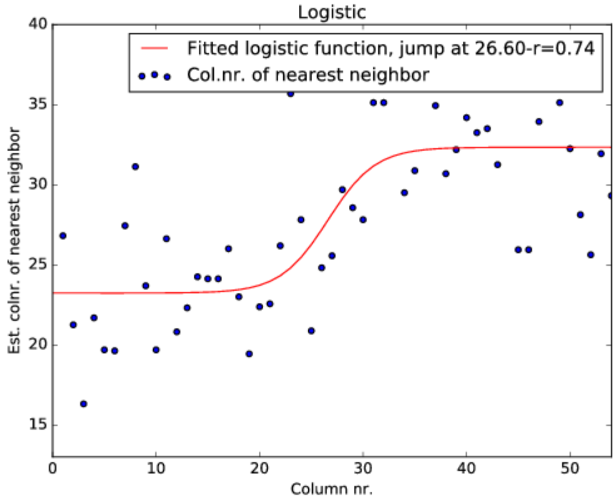

Fig 6. Average serial position of nearest-neighbour of a column in fraglet-feature space (raw values), with a least-squares fitted logistic curve.

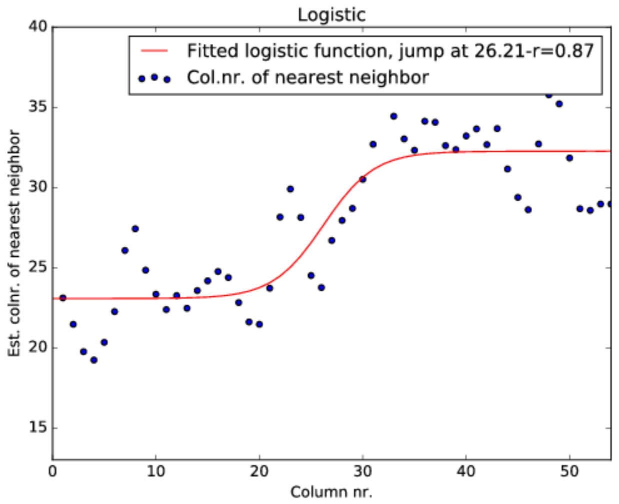

Fig 7. Average serial position of nearest-neighbour of a column in fraglet-feature space (smoothed over 3 values), with a least-squares fitted logistic curve.

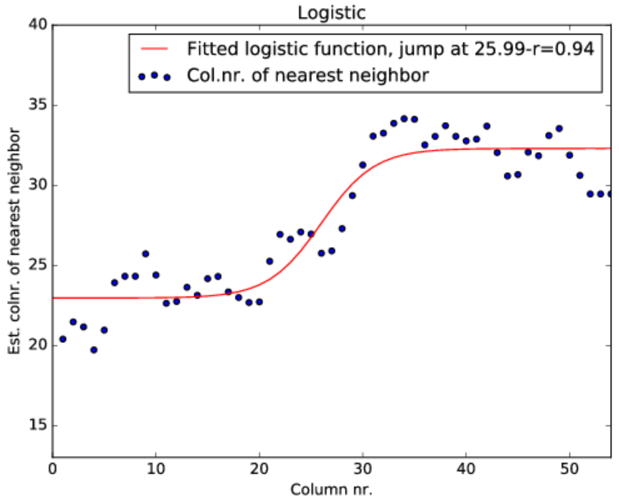

Fig 8. Average serial position of nearest-neighbour of a column in fraglet-feature space (smoothed over 5 values), with a least-squares fitted logistic curve.

S5 Tertiary analyses

The charts with full character shapes for individual Hebrew letters improve significantly on the traditional palaeographic chart. Each instance of a character can be directly traced back to its exact position in the manuscript of 1QIsa[a]. Also, there is no modern human hand involved, either in retracing the characters or in character reconstruction. The ink traces are extracted as is from the digital images and retain the movements once made by the ancient scribe's hand. Figure 9 presents two such charts. It is possible to request charts from all the individual characters by emailing the corresponding author.

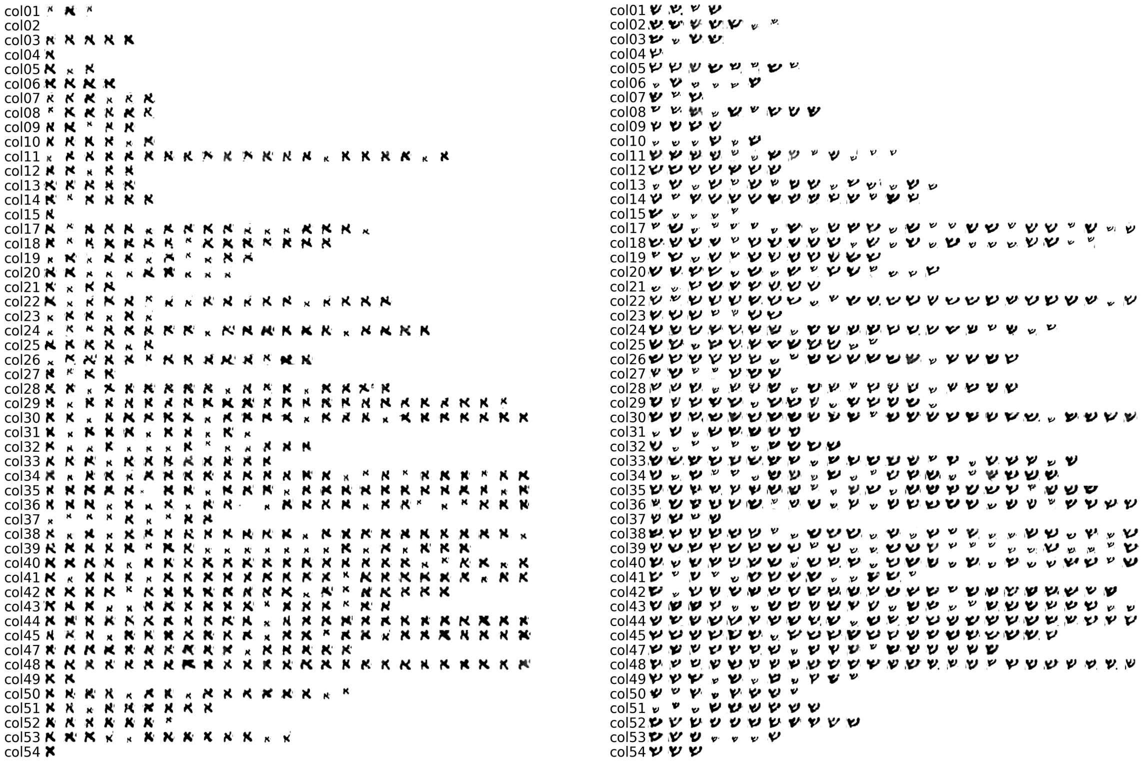

Fig 9. Individual character shapes of *aleph* (left) and *shin* (right) extracted from each of the columns of 1QIsa[a].

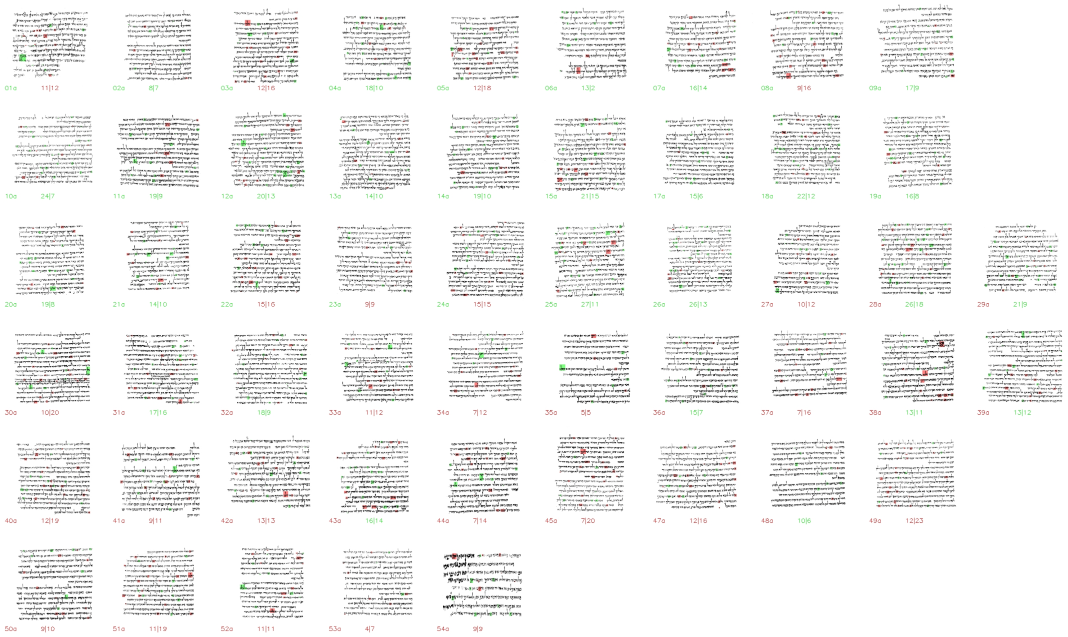

Fig 10. Visually enhanced presence of typical 'left' fraglets (green) and 'right' fraglets, separately for the 'a' split scans (top-halves) of the columns.

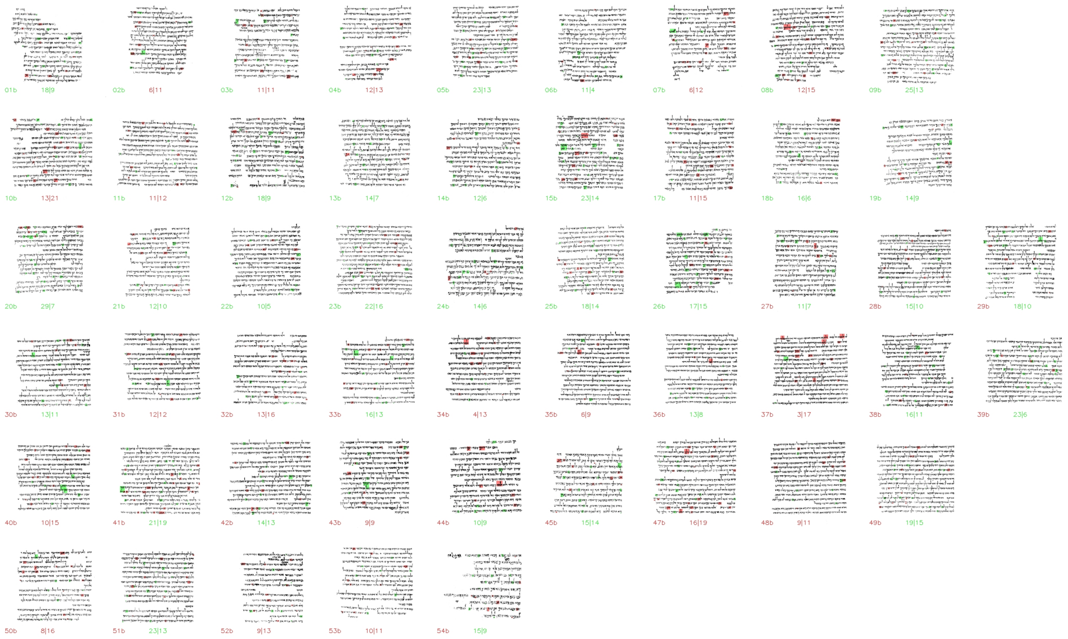

Fig 11. Visually enhanced presence of typical 'left' fraglets (green) and 'right' fraglets, separately for the 'b' split scans (bottom-halves) of the columns.

References

1. Ulrich E, Flint PW. Discoveries in the Judaean Desert XXXII: Qumran Cave 1: II. The Isaiah Scrolls: Part 2: Introductions, Commentary, and Textual Variants. OUP Oxford; 2011.

2. Tov E. Scribal practices and approaches reflected in the texts found in the Judean Desert. Brill; 2004.

3. Otsu N. A threshold selection method from gray-level histograms. IEEE transactions on systems, man, and cybernetics. 1979;9(1):62–66.

4. Sauvola J, Pietikäinen M. Adaptive document image binarization. Pattern recognition. 2000;33(2):225–236.

5. Dhali MA, de Wit JW, Schomaker L. BiNet: Degraded-Manuscript Binarization in Diverse Document Textures and Layouts using Deep Encoder-Decoder Networks. arXiv preprint arXiv:191107930. 2019;.

6. Team TGD. Gnu image manipulation program - GIMP - version 2.8.6; 2016.

7. Bulacu M, Brink A, van der Zant T, Schomaker L. Recognition of Handwritten Numerical Fields in a Large Single-Writer Historical Collection. In: 2009 10th International Conference on Document Analysis and Recognition; 2009. p. 808–812. Available from: `https://github.com/GrHound/imagemorph.c`.

8. Bulacu M, Schomaker L. Text-independent writer identification and verification using textural and allographic features. IEEE transactions on pattern analysis and machine intelligence. 2007;29(4):701–717.

9. The Leon Levy Dead Sea Scrolls Digital Library;. `https://www.deadseascrolls.org.il/`.

10. Dhali MA, He S, Popović M, Tigchelaar E, Schomaker L. A digital palaeographic approach towards writer identification in the dead sea scrolls. In: Proceedings of the 6th International Conference on Pattern Recognition Applications and Methods-Volume 1: ICPRAM. vol. 2017. Scitepress; Setúbal; 2017. p. 693–702.

11. Dillon M. Introduction to modern information retrieval: G. Salton and M. McGill.; 1983.
