# Supplementary material for: Artificial intelligence based writer identification generates new evidence for the unknown scribes of the Dead Sea Scrolls exemplified by the Great Isaiah Scroll (1QIsaa)
Source: PLoS One. 2021 Apr 21;16(4):e0249769. doi: 10.1371/journal.pone.0249769 (PMC8059865; doi:10.1371/journal.pone.0249769)
Supplement: Attachment — Submitted filename: Response to Reviewers.pdf [file pone.0249769.s002.pdf]

**Rebuttal letter**

*Artificial intelligence based writer identification generates new evidence for the unknown scribes of the Dead Sea Scrolls exemplified by the Great Isaiah Scroll (1QIsa[a]), Mladen Popović, Maruf A. Dhali, Lambert Schomaker*

We are very grateful to the academic editor and the reviewers for their careful assessment and we are pleased with the very positive reviews and valuable feedback, which is in line with our own approach and thinking on these issues.

The reviewers recognize and appreciate our innovative approach to palaeographic writer identification using AI machine learning, statistics, and data visualizations. Both reviewers acknowledge that our study is technically sound, and that the data support the conclusions. Both affirm that our article describes a technically sound piece of scientific research with data that supports the conclusions. The reviewers agree that our statistical analysis have been performed appropriately and rigorously. Both concur that we made all data underlying the findings in our manuscript fully available. And they agree that our manuscript is presented in an intelligible fashion and written in standard English.

We thank the academic editor and the reviewers for their feedback which has helped to further clarify our study and improve on our article.

This rebuttal letter responds to each point raised by the academic editor and reviewers. We first give our answers to the reviewers' comments and then our answers to the editorial remarks regarding journal requirements. We hope that this rebuttal letter and our revisions to the article have sufficiently clarified and resolved the points raised.

**A. Reviewers' comments:**

We thank reviewer #1 for the very positive assessment of our approach to the writer identification process being a machine-based and statistical one, rather than just the opinion of a human analyst, indicating two writers.

We thank reviewer #2 for the appreciation of the significance of our work and of us advancing palaeography by our data visualizations. Regarding the reviewer's suggestion to release a data visualization tool, we have indeed thought about this. In the future we hope to make a web-based application available for experts and students. For the publication now, we have created a Zenodo DOI link where we have uploaded the binarized images, feature files, and a script for 3D visualization for the point clouds in PCA space. We refer to this at the end of the Methods and materials section.

Regarding the further points of feedback raised by reviewer #2, which are in line with our own approach and thinking on these issues:

1. We are grateful for the acknowledgement of the significance of our methodology to palaeography. We also appreciate that the comments of reviewer #2 have contributed to further strengthening our conclusions. After performing additional analysis, including random elastic morphing on the column images and an additional application of the logistic model to another shape feature than the Fraglets (i.e., now also to the Hinge feature), the presence of two statistically different signal sources in the column series appears to be clear.

2. We are pleased with the reviewer's acknowledgement that in a sound and scientific manner our experiment shows that two scribes can be argued for. Together with his further points 3-5 we have performed additional tests that have further demonstrated the robustness of our method and conclusion.

We have clarified in the introduction that in scrolls scholarship it is far from there being a recognition in the field that both positions are possible from a palaeographic point of view. On the contrary, hardly any palaeographic substantiation whatsoever has been put forward, except for Ulrich/Flint. In terms of palaeography, we have found a new means to move forward and present new evidence for two scribes, and we are pleased with reviewer #2's appreciation of this.

3. We fully agree with the line of thinking of reviewer #2. This is exactly why we perform our analyses and tests, to find out what best explains the evidence for a clear break.

While it is correct that there is variability in character shapes even when there is one scribe this does not mean that the overall data is best explained by within-writer variability. One would then expect the point cloud distribution to be more random instead of two separate clusters, without points going into each other's area.

Also, the change-of-pen-argument is weakened because Hinge and Fraglet analyses tap into different sources of information (Fraglets contain the larger, complicated character fragments, while the Hinge feature concerns local curvature) and both methods point to a clear break in the data and separation of two clusters.

Moreover, we have performed an additional statistical F test that demonstrates a difference of variances of distance such that the variance is higher in the second part of the manuscript. This is indicative of more variable writing patterns in columns 28-54 over against 1-27.

So, although one cannot rule out completely that the clear separation between the two halves of the manuscript and the difference in variance of writing patterns in each half is because of a change of writing implement (a different pen), writing fatigue or some injury that the writer suffered when moving on to the second half of the manuscript, the more straightforward explanation is that a change in writers occurred.

We have further clarified this in the Results section, and in the Discussion and conclusions section.

We would love to perform the additional test the reviewer asks for, but this cannot be performed on another text from the scrolls with a known author because the authors are not known; this is exactly the outstanding issue in the field we have advanced through our innovative approach. However, all our tests have inherently performed verifications for a known writer if someone reluctantly considers that only the first half is written by one scribe and then ignores the second half. Even after

adding noise (new tests in the revision phase), the writers' clusters still hold, and the separability between writers still prevails. This outcome also cross-validates the system's robustness of writer identification. Nevertheless, we will perform our analysis on other texts from the scrolls in the near future, but those are separate studies. Of course, our writer identification tools have been validated by other corpora, to which we refer (see the 'Firemaker' reference on p. 20).

4. Again, we completely agree with reviewer #2. This is why we have performed additional stress tests adding noise in the form of random elastic morphing, which show exactly that what the reviewer requests: the results are invariant/insensitive to the noise introduced in the data, they stay the same, demonstrating the robustness of our initial findings. We describe and clarify this in the Methods and materials section, in the Results section, and in the Discussion and conclusions section. We have added a GitHub link to the random morphing software.

5. Our additional tests have demonstrated the robustness of our approach and our results, confirming our conclusions regarding the data. See our answer above to point 4. The bistable configuration of writing styles is confirmed by an additional fit of the logistic model on the randomly morphed column images, in the original feature space (Fraglets), but now also for the Hinge and the Adjoined Hinge+Fraglets feature vector. All analyses confirm the presence of a switch point. For Fraglets the position of the switch point was confirmed, for the Hinge feature, it is estimated to occur a bit more to the right, but in any case with a high reliability (high r, low sigma).

6. While it is certainly true that not all tools used are necessarily AI-inspired, we believe that it is warranted to use "artificial intelligence" in the title and abstract to characterize our innovative digital palaeography approach as an overall AI-inspired approach:

- The BiNet algorithm for binarization is a Convolutional Neural Network, in other words deep learning
- The Hinge approaches are from Pattern Recognition, generally considered as a subdiscipline in AI
- The codebook approaches (Kohonen maps) are from AI
- This is AI in the sense that we wanted to minimize human influence, letting the data speak
- In the classification stage we avoided deep learning to avoid discussions concerning explainability

We have clarified this accordingly in the Materials and methods section.

**B. Editorial comments regarding journal requirements:**

1. We have done so accordingly, we have used the PLOS ONE style template (LaTeX).

2. We have explicated the reference on p. 17 and also on p. 15.

3. We have taken up a reference to the table on p. 5.

4. We have received from Suzanne Mekking, Senior Acquisitions Editor Old Testament and Qumran, from Brill Publishers, the original copyright holder, the completed Content Permission Form for publication of the original image and attach it to this revised submission.

We have also included the text in the figure caption of the copyrighted figure on p. 6.
